# Association between ABCB1 polymorphisms and haplotypes and Alzheimer’s disease: a meta-analysis

**DOI:** 10.1038/srep32708

**Published:** 2016-09-07

**Authors:** Xin Zhong, Ming-Yan Liu, Xiao-Hong Sun, Min-Jie Wei

**Affiliations:** 1 School of Pharmacy, Department of Pharmacology, China Medical University, Shenyang, Liaoning 110122, China; 2 Department of Neurology, the Fourth Affiliated Hospital of China Medical University, Shenyang, Liaoning 110032, China

## Abstract

Although several epidemiological studies have investigated the association between ATP-binding cassette subfamily B member 1 (ABCB1) gene polymorphisms and Alzheimer’s disease (AD) susceptibility, controversial results exist. Here, we performed a meta-analysis to assess whether ABCB1 polymorphisms 3435C > T (rs1045642), 2677G > T/A (rs2032582), 1236C > T (rs1128503) and haplotypes were associated with AD risk. Nine independent publications were included and analyzed. Crude odds ratio (OR) and 95% confidence interval (CI) were applied to investigate the strength of the association. Sensitivity analysis was conducted to measure the robustness of our analysis. A funnel plot and trim and fill method were used to test and adjust for publication bias. The results showed a significant association between the 3435C > T single nucleotide polymorphism (SNP) and AD susceptibility (CT vs. CC: OR = 1.24, 95% CI = 1.06–1.45, *P* = 0.01; CT + TT vs. CC: OR = 1.21, 95% CI = 1.04–1.41, *P* = 0.01) in the total population, as well as in Caucasian subgroup. The 2677G > T/A SNP was related to a decreased AD risk in Caucasian subgroup (TT + TA + AA vs. GT + GA + GG: OR = 0.68, 95% CI = 0.47–0.98, *P* = 0.04). Moreover, the ABCB1 haplotype analysis showed that the 1236T/2677T/3435C haplotype was associated with a higher risk of AD (OR = 1.99, 95% CI = 1.24–3.18, *P* = 0.00). Our results suggest that the ABCB1 3435C > T SN*P*, the 2677G > T/A SNP and 1236T/2677T/3435C haplotype are significantly associated with AD susceptibility.

Alzheimer’s disease (AD) is the most common progressive neurodegenerative disorder of the central nervous system, clinically characterized by memory impairment and cognitive dysfunction. The pathological hallmarks of AD in the brain are massive cerebral accumulation of senile plaques largely composed of β-amyloid (Aβ) peptide, intracellular neurofibrillary tangles and neuronal loss^1^,^2^,^3^,^4^. Recently, several epidemiological studies have revealed that mutations of particular genes can confer susceptibility to the pathogenesis of AD. APP, PSEN1, PSEN2 gene mutations are considered to be the main causes of familial AD, and play an important role in the early-onset of AD^5^. While in late-onset AD, the ε4 allele of the Apolipoprotein E gene (ApoEε4) is known as the major genetic risk factor^6^. Moreover, the mutations of genes such as APBB2, GAB2, PRNP and SORL1 have also been reported to be associated with the risk of AD^7^,^8^,^9^,^10^,^11^.

The ATP-binding cassette subfamily B member 1 (ABCB1) gene in the chromosome 7q21 region spans 209-kb and contains 29 exons^12^. To date, several ABCB1 genetic variants have been identified to be involved in various kinds of diseases through re-sequencing, including 3435C > T (rs1045642), 2677G > T/A (rs2032582), 1236C > T (rs1128503) polymorphisms. The 3435C > T single nucleotide polymorphism (SNP) was the most commonly studied polymorphism, which was previously reported to be related to the susceptibility to the osteonecrosis of the femoral head (ONFH), leukemia, breast cancer and hepatocellular carcinoma^13^,^14^,^15^,^16^. The 2677G > T/A polymorphism is associated with glucocorticoid-induced avascular necrosis of the femoral head (GANFH) and cancer^17^,^18^. The 1236C > T polymorphism may contribute to the tumor response to chemotherapy in cancers in Asians^19^,^20^.

P-glycoprotein (P-gp) is encoded by the ABCB1 gene and acts as an integral component of the blood-brain barrier (BBB)^21^. P-gp pumps various drugs and toxicants out of the brain and Aβ is an endogenous substrate for P-gp, suggesting that P-gp is able to mediate the clearance of Aβ through BBB^22^. Therefore, mutations of ABCB1 gene possibly result in an aberrant function of P-gp, and thus promote the pathogenesis of AD. Recently, some epidemiological studies have focused on the association between ABCB1 gene variants and AD susceptibility, and these variants include the 3435C > T polymorphism, the 2677G > T/A polymorphism and the 1236C > T polymorphism^23^,^24^,^25^,^26^,^27^,^28^,^29^,^30^,^31^. The 3435C > T polymorphism of ABCB1, which is a C to T transformation in exon 26 with no change in the amino acid encoded, is not related to AD risk in most studies^24^,^25^,^27^,^29^,^30^,^31^. However, Fehér *et al*.^23^ and van Assema *et al*.^26^ suggested a positive association between the 3435C > T polymorphism and AD susceptibility. The 2677G > T/A SNP is a missense mutation in exon 21, leading to an alanine to threonine or serine substitution. The association between the 2677G > T/A SNP and the risk of AD is also controversial^23^,^24^,^26^,^27^,^31^. The ABCB1 1236C > T polymorphism is a nucleotide change in exon 12 with no change in the amino acid encoded, and van Assema *et al*.^26^ suggested 1236C > T polymorphism might contribute to the progression of Aβ deposition in brain. Because of multiple sites in linkage disequilibrium, haplotypes analysis is necessary to consider the impact of genetic information and guarantee the risk assessment process. In this study, we carried out a meta-analysis to investigate the associations between ABCB1 gene polymorphisms and haplotypes and AD risk.

## Results

### Characteristics of eligible studies

**Figure 1:**
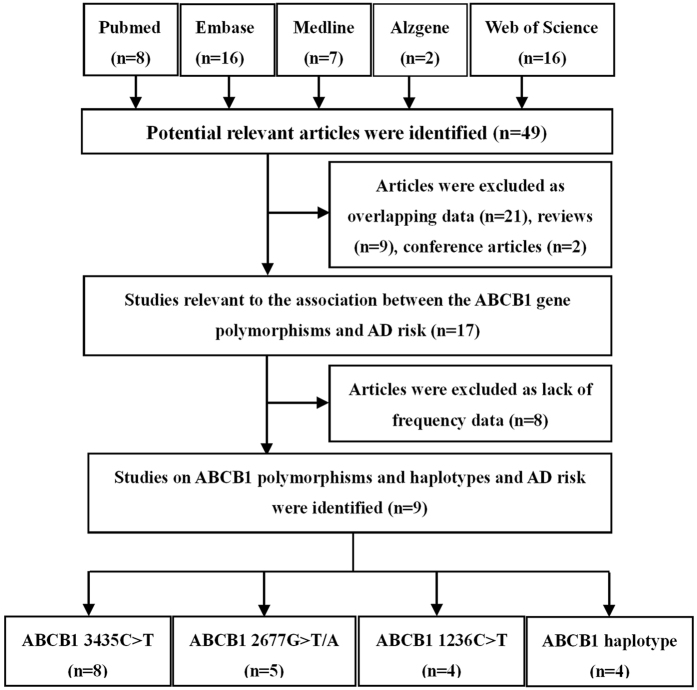
Flow diagram of search strategy and study selection for meta-analysis.

Fourty-nine potential studies were identified after an initial search from the Pubmed, Embase, Web of Science, Medline and Alzgene databases. After further screening, nine studies were finally enrolled in our meta-analysis (2366 cases and 2256 controls). The flow diagram of the search process was shown in Fig. 1. The ethnicities of the populations involved in these studies were Caucasian, Asian and mixed. AD was diagnosed according to the criteria set by the Diagnostic and Statistical Manual of Mental Disorders (DSM) or National Institute of Neurological and Communicative Disorders and Stroke and the Alzheimer’s Disease and Related Disorders Association (NINCDS–ADRDA)^32^. Most studies used the Mini Mental State Examination (MMSE) to identify healthy controls^33^. The median age of AD cases in these studies ranged from 64 to 82.5 years and the percentage of female patients ranged from 29.4 ~ 74.1%. The methods for gene identification consisted of the Polymerase Chain Reaction-Restriction Fragment Length Polymorphism (PCR-RFLP), a direct sequencing method and the TaqMan assay. Three common polymorphisms in the ABCB1 gene (3435C > T, 2677G > T/A, 1236C > T) and three haplotypes (1236T/2677T/3435T, 1236T/2677T/3435C, 1236C/2677T/3435T) were assessed. The genotype frequencies of controls were all in Hardy-Weinberg Equilibrium (HWE) (*P* > 0.05). The key information of the studies on ABCB1 3435C > T, 2677G > T/A, 1236C > T polymorphisms and AD risk is summarized in Table 1. The Magliulo’s study^28^ was not included in Table 1, because this study only performed the ABCB1 haplotype analysis.

### Meta-analysis: ABCB1 3435C > T SNP

**Figure 2:**
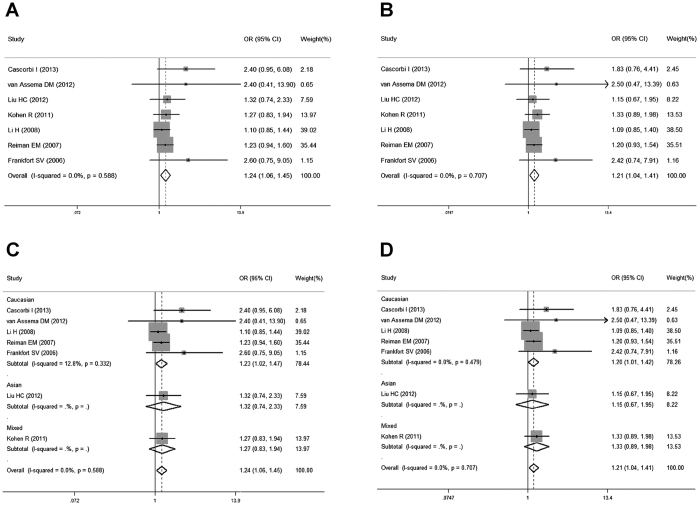
Forest plots for the association between the ABCB1 3435C > T SNP and AD risk. (**A**) heterozygous comparison model for overall populations; (**B**) dominant model for overall populations; (**C**) heterozygous comparison model for ethnicity subgroup; (**D**) dominant model for ethnicity subgroup.

Eight studies investigated the association between the 3435C > T SNP and AD susceptibility. The pooled summary crude odds ratios (ORs) and corresponding 95% confidence intervals (CIs) in all the genetic models were as follows: TT vs. CC: OR = 1.04, 95% CI = 0.76–1.41, *P* = 0.82; CT vs. CC: OR  =  1.18, 95% CI = 0.97–1.45, *P* = 0.10; CT + TT vs. CC: OR = 1.14, 95% CI = 0.91–1.42, *P* = 0.26; TT vs. CT + CC: OR = 0.91, 95% CI = 0.75–1.11, *P* = 0.36; T vs. C: OR = 1.01, 95% CI = 0.87–1.17, *P* = 0.90. A moderate heterogeneity was found between the individual studies (TT vs. CC: χ^2^ = 15.51, *P* = 0.03, I^2^ = 54.9%; CT vs.CC: χ^2^ = 9.56, *P* = 0.22, I^2^ = 26.8%; CT + TT vs. CC: χ^2^ = 12.5, *P* = 0.09, I^2^ = 44%; TT vs. CT + CC: χ^2^ = 11.18, *P* = 0.13, I^2^ = 37.4%; T vs. C: χ^2^ = 14.64, *P* = 0.04, I^2^ = 52.2%). We conducted a sensitivity analysis by excluding each study, and found the Fehér’s study^23^ could interpret the heterogeneity, because the statistical power of this study was higher than the others (OR > 1.25, P < 0.05). Moreover, after the exclusion of the Fehér’s study^23^, the I^2^ decreased to 0% with a different but statistically significant pooled estimate in the homozygote comparison model (CT vs. CC: OR = 1.24, 95% CI = 1.06–1.45, *P* = 0.01, Fig. 2A) and the dominant model (CT + TT vs. CC: OR = 1.21, 95% CI = 1.04–1.41, *P* = 0.01, Fig. 2B), indicating that the Fehér’s study^23^ was not robust. These results suggested that there might be an association between ABCB1 3435C > T SNP and AD susceptibility.

Additionally, subgroup analysis by ethnicity showed a significant association between 3435C > T SNP and an increased risk of AD among Caucasians (CT vs.CC: OR = 1.23, 95% CI = 1.02–1.47, *P* = 0.03, Fig. 2C; CT + TT vs. CC: OR = 1.20, 95% CI = 1.01–1.42, *P* = 0.04, Fig. 2D), but not among Asians or mixed. Pooled ORs and 95% CIs for the association of ABCB1 3435C > T SNP with AD risk are shown in Table 2.

**Figure 3:**
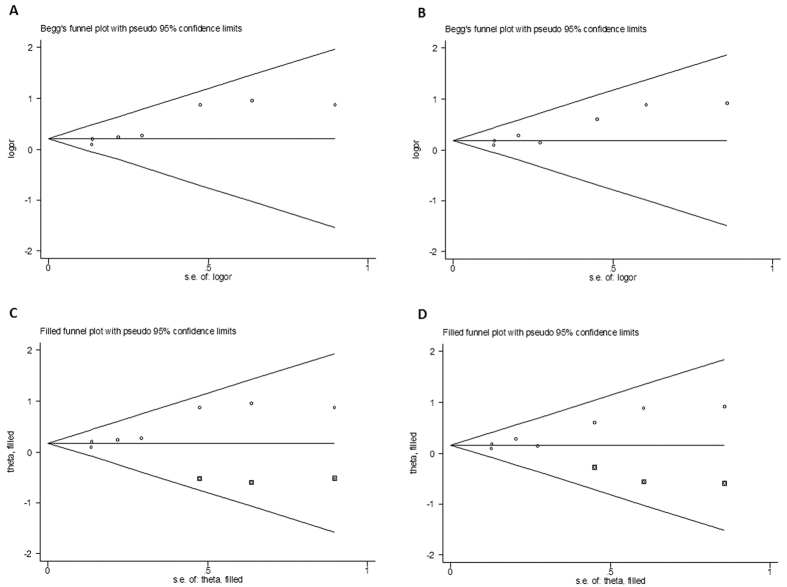
Funnel plot analysis and the trim and fill method to detect publication bias of ABCB1 3435C > T SNP. (**A**) funnel plot analysis in heterozygous comparison model; (**B**) funnel plot analysis in dominant model; (**C**) trim and fill method in heterozygous comparison model; (**D**) trim and fill method in dominant model. Circles represent the weight of the studies and boxes stand for the added studies.

Funnel plots were adopted to assess the publication bias, and an evidence of asymmetry was observed (CT vs.CC: *P* = 0.04, Fig. 3A; CT + TT vs. CC: *P* = 0.07, Fig. 3B). This result was further supported by the analysis using Egger’s test (TT vs.CC: *P* = 0.28; CT vs.CC: *P* = 0.01; CT + TT vs. CC: *P* = 0.01; TT vs. CT + CC: *P* = 0.61; T vs. C: *P* = 0.39). However, the funnel plots after the adoption of trim and fill method had perfect symmetry and no significant difference was found between the ORs and 95% CIs before and after, suggesting that there were no change within the crude and adjusted estimates (Fig. 3C,D for CT vs. CC model and CT + TT vs. CC model, respectively). These findings indicated that the meta-estimates for 3435C > T SNP were robust to confounders and methods of exposure assessment. Therefore, there appeared to be an association between the ABCB1 3435C > T SNP and an increased AD risk according to our analysis.

### Meta-analysis: ABCB1 2677G > T/A SNP

**Figure 4:**
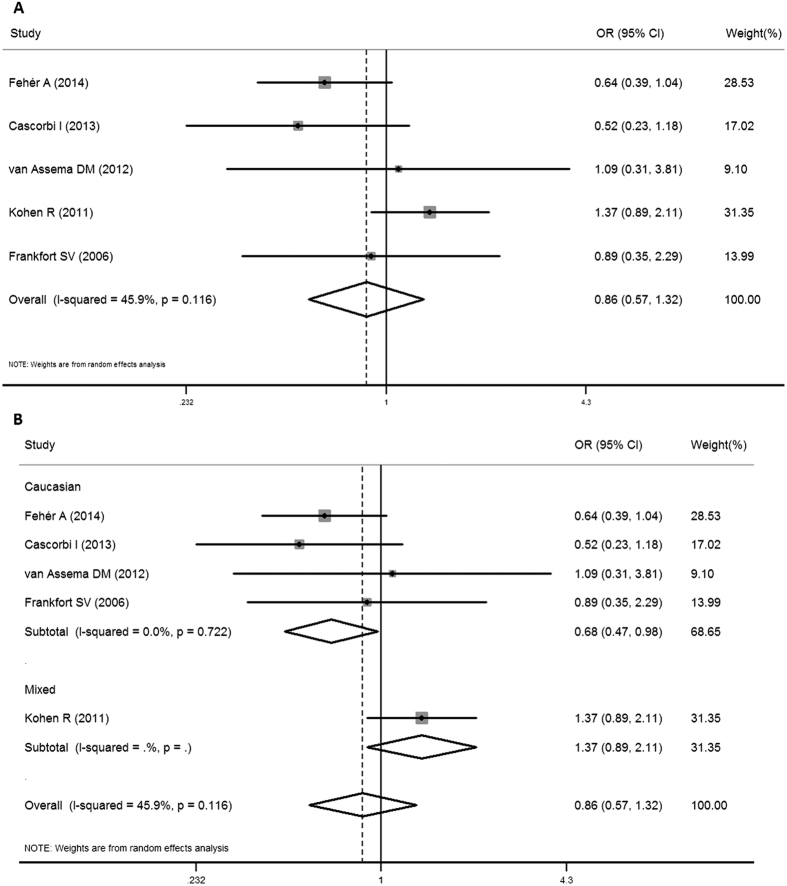
Forest plots for the association between ABCB1 2677G > T/A SNP and AD risk in the recessive model. (**A**) for overall populations; (**B**) for ethnicity subgroup.

**Figure 5:**
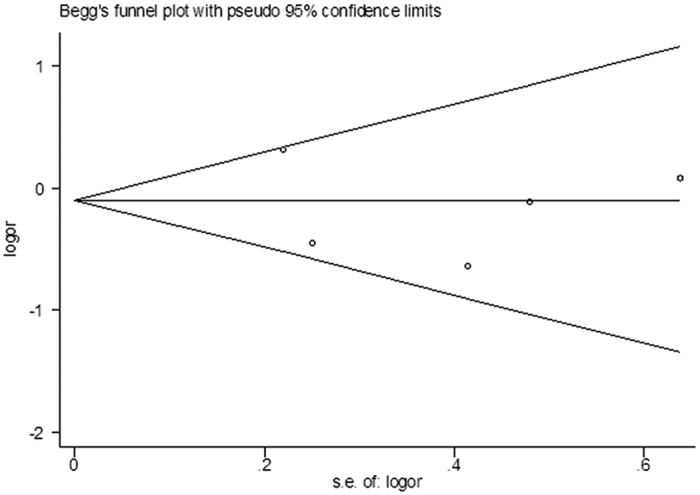
Funnel plot analysis to detect publication bias of ABCB1 2677G > T/A SNP in the recessive model.

Five studies investigated the association of 2677G > T/A SNP with the AD risk. No significant association between 2677G > T/A SNP and AD susceptibility was detected in the total populations (for TT + AA vs. GG: OR = 0.99, 95% CI = 0.59–1.67, *P* = 0.97, for GT + GA vs. GG: OR = 1.20, 95% CI  = 0.93–1.56, *P* = 0.16, for GT + TT + GA + AA + TA vs. GG: OR = 1.15, 95% CI  = 0.90–1.46, *P* = 0.27, for TT + TA + AA vs. GT + GA + GG: OR = 0.86, 95% CI  = 0.57–1.32, *P* = 0.50, Fig. 4A, for T + A vs. G: OR = 1.01, 95% CI = 0.80–1.29, *P* = 0.91). An obvious heterogeneity was observed in most models (for TT + AA vs. GG: χ^2^ = 8.29, *P* = 0.08, I^2^ = 51.8%, for GT + GA vs. GG: χ^2^ = 1.89, *P* = 0.76, I^2^ = 0.0%, for GT + TT + GA + AA + TA vs. GG: χ^2^ = 3.72, *P* = 0.45, I^2^ = 0.0%, for TT + TA + AA vs. GT + GA + GG: χ^2^ = 7.4, *P* = 0.12, I^2^ = 45.9%, for T + A vs. G: χ^2^ = 7.21, *P* = 0.13, I^2^ = 44.6%). However, subgroup analysis according to the ethnicity in the recessive model showed a significant association between 2677G > T/A SNP and a decreased AD risk among Caucasians (TT + TA + AA vs. GT + GA + GG: OR = 0.68, 95% CI  = 0.47–0.98, *P* = 0.04) with no heterogeneity (χ^2^ = 1.33, *P* = 0.72, I^2^ = 0.0%), but not in mixed (Fig. 4B). Begg’s funnel plot and Egger’s linear regression test were used to assess the publication bias. All *P* values of Begg’s funnel plots were more than 0.05 (for TT + AA vs. GG: *P* = 0.81, for GT + GA vs. GG: *P* = 0.46, for GT + TT + GA + AA + TA vs. GG: *P* = 0.81, for TT + TA + AA vs. GT + GA + GG: *P* = 0.81, for T + A vs. G: *P* = 0.81), as well as the Egger’s linear regression test (for TT + AA vs. GG: *P* = 0.92, for GT + GA vs. GG: *P* = 0.27, for GT + TT + GA + AA + TA vs. GG: *P* = 0.66, for TT + TA + AA vs. GT + GA + GG: *P* = 0.64, for T + A vs. G: *P* = 0.97), suggesting that there was no publication bias in this meta-analysis. Pooled ORs and 95% CIs for the association of ABCB1 2677G > T/A SNP with AD risk are shown in Table 2, and the funnel plot for the recessive model is demonstrated in Fig. 5.

### Meta-analysis: ABCB1 1236C > T SNP

Four studies were selected to estimate the association of the ABCB1 1236C > T SNP with the risk of AD. As a result, no statistically significant difference was found in all the genetic models. Subgroup analysis for the 1236C > T SNP showed no significant differences in overall ORs (Table 2).

### Meta-analysis: ABCB1 haplotypes

Four studies investigated the role of ABCB1 haplotypes in AD, including the 1236T/2677T/3435T (TTT) haplotype, the 1236T/2677T/3435C (TTC) haplotype and the 1236C/2677T/3435T (CTT) haplotype (Table 3). The pooling estimates showed the TTC genotype was associated with a significant increase in the risk of AD (OR = 1.99, 95% CI = 1.24–3.18, *P* = 0.00). The TTT and CTT haplotypes exhibited no association with the AD risk (CTT: OR = 0.58, 95% CI  = 0.31–1.08, *P* = 0.09; TTT: OR = 0.90, 95% CI  = 0.56–1.44, *P* = 0.65, Table 4).

## Discussion

As one of the most common forms of dementia, AD affects millions of people worldwide. Recently, several studies have revealed that mutations of ABCB1 can be responsible for the pathogenesis of AD by promoting the accumulation of Aβ via P-gp^22^,^23^,^24^,^26^. Although ABCB1 gene variants such as 3435C > T (rs1045642), 2677G > T/A (rs2032582), 1236C > T (rs1128503) polymorphisms have been reported to be associated with AD risk^23^,^24^,^26^, several researchers have shown opposite results^25^,^27^,^28^,^29^,^30^,^31^. To investigate if ABCB1 polymorphisms are associated with the susceptibility to AD, we conducted this meta-analysis to study the relationship between ABCB1 polymorphisms and AD risk.

Fehér *et al*.^23^ reported that the ABCB1 3435C > T SNP was associated with AD. Van Assema *et al*.^26^ supported that ABCB1 3435C > T, 2677G > T/A, 1236C > T SNPs were related to changes in P-gp function at the BBB in AD patients, and thus may contribute to the pathogenesis of AD. In this study, we found a positive association between the ABCB1 3435C > T polymorphism and AD susceptibility using a heterozygous comparison model and a dominant model. Sensitivity analysis was applied to ascertain the potential source of the heterogeneity between the studies. After excluding the Fehér’s study^23^ due to its high OR, homogeneity across studies was achieved, and a statistically significant association between the 3435C > T SNP and AD risk was found. Moreover, subgroup analysis by ethnicity showed a significant association between the 3435C > T SNP and an increased risk of AD among Caucasians, but not in Asians or mixed races. Trim and fill analysis for 3435C > T SNP meta-estimates demonstrated no evidence of publication bias in these studies, suggesting a definite association between the ABCB1 3435C > T SNP and AD risk.

Consistent with the report by Cascorbi *et al*.^24^, we found that there was an association between the ABCB1 2677G > T/A SNP and a decreased AD susceptibility among Caucasian, but not in total population, implying that the 2677G > T/A SNP may play a potential role in the AD pathogenesis. Moreover, our results demonstrated that there was no association between the ABCB1 1236C > T SNP and AD risk.

Due to the existence of multiple genes in linkage disequilibrium, haplotype analysis was useful to identify other indirect SNPs at the same region. As for the ABCB1 gene, the TTT, TTC and CTT haplotypes were most investigated for their roles in AD susceptibility. The studies by Fehér *et al*.^23^, Cascorbi *et al*.^24^ and Frankfort *et al*.^31^ supported that there were no associations between these haplotypes and AD risk. However, Magliulo *et al*.^28^ found that the TTT haplotype might play a role in donepezil disposition and clinical outcome. In the present study, we found no association between TTT, CTT haplotypes and the risk of AD, consistent with most studies^23^,^24^,^31^. In contrast, although no studies have identified a positive association between the TTC haplotype and AD risk, our meta-analysis showed the TTC haplotype was significantly associated with an increased AD susceptibility. In addition, no heterogeneity was found in the overall models, suggesting that the results of this meta-analysis were statistically robust.

A recent Genome-Wide Association Study (GWAS) by Lambert *et al*.^11^ found that no SNPs in the ABCB1 gene are genome-wide significant. In this study, we found that the 3435C > T SNP and 2677G > T/A SNP were significantly associated with AD risk. Different ethnicities may contribute to the difference between our study and the GWAS, because the GWAS consisted of individuals of European ancestry, while our study included Caucasians and Asians participants. Moreover, the GWAS focused on the possible susceptibility loci for late-onset AD, whereas our study evaluated the association between ABCB1 SNPs and early-onset and late-onset AD.

There are some limitations in this meta-analysis. First, although we collected all the eligible publications, studies included in our meta-analysis were still limited. Randomized controlled clinical trials with larger sample sizes are required to confirm our conclusion. Second, AD is a multifactorial disease, and the potential interactions among gene-gene and gene-environment should take into account. Many other factors such as ApoEε4 may participate in the association between ABCB1 polymorphisms and AD risk. However, we did not carry out a subgroup analysis based on it due to limited data. Third, since some studies have not provided informations such as age, gender, diagnostic criteria and sequencing method, we could not carry out a subgroup analysis to study the effects of these parameters on the association between ABCB1 polymorphisms and AD risk. To better understand the role of ABCB1 polymorphisms or haplotypes in AD susceptibility, large-scale, high-quality studies with multi-ethnic populations are needed.

In conclusion, based on the published studies, our meta-analysis confirmed that the ABCB1 3435C > T SNP and TTC haplotype were significantly associated with an increased AD risk, while the 2677G > T/A SNP may contribute to a lower susceptibility of AD. Our study, for the first time, found that there were associations between ABCB1 polymorphisms and haplotypes and AD risk. Our findings provide a better understanding of AD pathogenesis, and ABCB1 polymorphisms and haplotypes may serve as important targets for the prevention and diagnosis for AD.

## Methods

### Selection of studies

To identify studies eligible for the meta-analysis, a comprehensive search strategy was applied using the electronic databases including Pubmed, Embase, Web of Science, Medline and Alzgene. The following keywords were used: ABCB1 or ATP-binding cassette subfamily B member 1, and polymorphism or rs1045642 or rs2032582 or rs1128503, and Alzheimer’s disease or AD. All selected articles were published from January 2001 to March 2016. Other relevant studies were identified by hand searching the references of included articles.

### Data extraction

Studies included in this meta-analysis met the following criteria: (1) the study had to be focused on the 3435C > T, 2677G > T/A, 1236C > T SNP or haplotypes within the ABCB1 gene using original data; (2) the study should be case-control-designed or cohort-designed; (3) all patients must meet the diagnostic criteria of AD; (4) both AD patients and controls were included; and (5) the frequencies of individual genotypes in cases and controls were reported or provided from the authors. Exclusion criteria included studies with no relevance to ABCB1 polymorphisms and AD risk, overlapping data, and review articles. Each article was checked with inclusion and exclusion criteria independently by two investigators (X.Z. and X.-H.S.).

### Statistical analysis

To assess the association between ABCB1 3435C > T, 2677G > T/A, 1236C > T polymorphisms and AD risk, ORs and corresponding 95% CIs were calculated using homozygote comparison (BB vs. AA), heterozygous comparison (AB vs. AA), dominant model (BB + AB vs. AA), recessive model (BB vs. AB + AA), and allele comparison (B vs. A) (A stands for major allele, B stands for minor allele). The significance of the pooled OR was determined by Z-test, and *P* < 0.05 was considered as statistically significant. We adopted the random-effects model to calculate the pooled ORs when a significant heterogeneity existed in the genetic models in the initial overall analysis, otherwise a fixed-effects model should be applied. The between-study heterogeneity was estimated using Cochran’s Q statistic and the I^2^ statistic. *P* > 0.10 in Q-test^34^ and I^2^ < 25%^35^ indicated no heterogeneity among studies. The HWE in the controls was measured by the Pearson’s Chi-square test, and the significance level was maintained at *P* < 0.05^36^. We assessed publication bias by Begg’s funnel plot and Egger’s linear regression test^37^, and adjusted for possible publication bias by means of the trim and fill method^38^. Moreover, subgroup analysis was conducted to explain the high heterogeneity in the studies according to the ethnicity. All statistical tests were performed using STATA 12.0 software (Stata Corporation, College Station, TX, USA).

## Additional Information

**How to cite this article**: Zhong, X. *et al*. Association between ABCB1 polymorphisms and haplotypes and Alzheimer’s disease: a meta-analysis. *Sci. Rep*. **6**, 32708; doi: 10.1038/srep32708 (2016).

## Figures and Tables

**Table 1:** Characteristics of individual studies on ABCB1 3435C > T, 2677G > T/A, 1236C > T polymorphisms and AD risk included in the meta-analysis.

First Author	Year	Country	Ethnicity	Diagnostic criteria	MRI/CT	MMSE	Genotyping	Sample size	Mean age	Female (%)	Case			Control			P_HWE_
3435C > T (rs1045642)								case/control	case/control	case/control	CC	CT	TT	CC	CT	TT	
Fehér, Á.	2014	Hungary	Caucasian	NINCDS/ADRDA	No	Yes	PCR-RFLP	234/225	75.6 ± 6.8/74.8 ± 7.2	67.9/66.7	66	119	49	43	109	73	0.84
Cascorbi, I.	2013	Germany	Caucasian	—	No	No	PCR-RFLP	71/81	78.4 ± 10.7/69.5 ± 11.4	54.9/40.7	9	42	20	17	33	31	0.15
Van Assema, D.M.	2012	Dutch	Caucasian	NINCDS/ADRDA	Yes	Yes	PCR-RFLP	32/17	64 ± 7/47 ± 17	29.4/43.8	2	9	6	8	15	9	0.73
Liu, H.C.	2012	China	Asian	—	No	No	—	111/114	82.5 ± 8.4/77.3 ± 6.6	63.1/52.6	44	52	15	49	44	21	0.06
Kohen, R.	2011	USA	Mixed	NINCDS/ADRDA	No	Yes	TaqMan	286/240	68.2 ± 9.4/52.5 ± 19.5	48/50	63	138	83	66	114	60	0.44
Li, H.	2008	Canada	Caucasian	DSM-IV	No	Yes	Sequencing	753/736	77.8 ± 8.6/73.4 ± 7.9	57.6/64.4	158	352	185	168	339	183	0.66
Reiman, E.M.	2007	USA/Netherlands	Caucasian	—	No	No	Sequencing	861/550	74.9 ± 6.6/77.4 ± 7.3	—	188	426	238	139	257	152	0.15
Frankfort, S.V.	2006	Netherlands	Caucasian	NINCDS/ADRDA	Yes	Yes	Sequencing	48/41	81 ± 5.5/81.9 ± 5.7	68.8/65.9	5	26	17	9	18	14	0.49
**2677G** > **T/A**(**rs2032582**)											**GG**	**GT** + **GA**	**TT** + **TA** + **AA**	**G/G**	**GT** + **GA**	**TT** + **TA** + **AA**	
Fehér, Á.	2014	Hungary	Caucasian	NINCDS/ADRDA	No	Yes	PCR-RFLP	234/225	75.6 ± 6.8/74.8 ± 7.2	67.9/66.7	69	132	33	62	117	46	0.50
Cascorbi, I.	2013	Germany	Caucasian	—	No	No	PCR-RFLP	71/81	78.4 ± 10.7/69.5 ± 11.4	54.9/40.7	25	35	11	27	33	21	0.10
Van Assema, D.M.	2012	Dutch	Caucasian	NINCDS/ADRDA	Yes	Yes	PCR-RFLP	32/17	64 ± 7/47 ± 17	29.4/43.8	3	8	6	10	10	10	0.07
Kohen, R.	2011	USA	Mixed	NINCDS/ADRDA	No	Yes	TaqMan	286/240	68.2 ± 9.4/52.5 ± 19.5	48/50	73	147	66	78	119	43	0.84
Frankfort, S.V.	2006	Netherlands	Caucasian	NINCDS/ADRDA	Yes	Yes	Sequencing	48/41	81 ± 5.5/81.9 ± 5.7	68.8/65.9	13	24	13	12	16	11	0.26
**1236C** > **T**(**rs1128503**)											**CC**	**CT**	**TT**	**CC**	**CT**	**TT**	
Fehér, Á.	2014	Hungary	Caucasian	NINCDS/ADRDA	No	Yes	PCR-RFLP	234/225	75.6 ± 6.8/74.8 ± 7.2	67.9/66.7	56	130	48	54	119	52	0.39
Cascorbi, I.	2013	Germany	Caucasian	—	No	No	PCR-RFLP	71/81	78.4 ± 10.7/69.5 ± 11.4	54.9/40.7	23	36	12	26	38	17	0.65
Van Assema, D.M.	2012	Dutch	Caucasian	NINCDS/ADRDA	Yes	Yes	PCR-RFLP	32/17	64 ± 7/47 ± 17	29.4/43.8	3	8	6	11	11	10	0.08
Frankfort, S.V.	2006	Netherlands	Caucasian	NINCDS/ADRDA	Yes	Yes	Sequencing	48/41	81 ± 5.5/81.9 ± 5.7	68.8/65.9	12	25	11	12	20	9	0.90

MRI/CT: Magnetic Resonance Imaging/Computerized Tomography; P_HWE_: P value of HWE in control.

**Table 2:** Meta-analysis of the ABCB1 3435C > T, 2677G > T/A, 1236C > T polymorphisms with AD risk.

3435C > T(rs1045642)	N	TT vs.CC				CT vs. CC				CT + TT vs. CC				TT vs. CT + CC				T vs. C			
		OR	95% CI	P	I^2^	OR	95% CI	P	I^2^	OR	95% CI	P	I^2^	OR	95% CI	P	I^2^	OR	95% CI	P	I^2^
Total	8	1.17	0.97–1.40	0.10	0	**1**.**24**	**1**.**06**–**1**.**45**	**0**.**01**	**0**	**1**.**21**	**1**.**04**–**1**.**41**	**0**.**01**	**0**	1.01	0.87–1.16	0.94	0	1.08	0.98–1.18	0.12	0
Ethnicity																					
Caucasian	5	1.15	0.94–1.41	0.17	0	**1**.**23**	**1**.**02**–**1**.**47**	**0**.**03**	**0**	**1**.**20**	**1**.**01**–**1**.**42**	**0**.**04**	**0**	0.99	0.84–1.16	0.89	0	1.06	0.96–1.17	0.26	0
Asian	2	0.80	0.37–1.73	0.56	0	1.32	0.74–2.33	0.35	0	1.15	0.67–1.95	0.61	0	0.69	0.34–1.42	0.32	0	0.97	0.66–1.42	0.86	0
Mixed	1	1.45	0.90–2.34	0.13	0	1.27	0.83–1.94	0.27	0	1.33	0.89–1.98	0.16	0	1.24	0.84–1.83	0.28	0	1.21	0.95–1.54	0.12	0
**2677G** > **T/A**(**rs2032582**)		**TT** + **AA vs**. **GG**				**GT** + **GA vs**. **GG**				**GT** + **TT** + **GA** + **AA** + **TA vs**. **GG**				**TT** + **TA** + **AA vs**. **GT** + **GA** + **GG**				**T** + **A vs**. **G**			
Total	5	0.99	0.59–1.67	0.97	51.8	1.20	0.93–1.56	0.16	0	1.15	0.90–1.46	0.27	0	0.86	0.57–1.32	0.50	45.9	1.01	0.80–1.29	0.91	44.6
Ethnicity																					
Caucasian	4	0.73	0.48–1.12	0.15	0	1.13	0.81–1.58	0.48	0	1.00	0.73–1.37	0.98	0	0.68	0.47–0.98	0.04	0	0.88	0.72–1.08	0.22	0
Mixed	1	1.64	1.00–2.70	0.05	0	1.32	0.88–1.97	0.17	0	1.41	0.96–2.05	0.08	0	1.37	0.89–2.11	0.15	0	1.28	1.00–1.63	0.05	0
**1236C** > **T**(**rs1128503**)		**TT vs**.**CC**				**CT vs**. **CC**				**CT** + **TT vs**. **CC**				**TT vs**. **CT** + **CC**				**T vs**. **C**			
Total	4	0.97	0.64–1.47	0.87	0	1.13	0.80–1.60	0.48	0	1.08	0.78–1.50	0.64	0	0.89	0.63–1.25	0.49	0	0.99	0.81–1.21	0.91	0

**Table 3:** Characteristics of studies on ABCB1 1236C > T-2677G > T/A-3435C > T haplotypes and AD risk included in the meta-analysis.

First Author	Year	Country	Ethnicity	Case	Control	Haplotype distribution					
						Case			Control		
						CTT	TTC	TTT	CTT	TTC	TTT
Fehér, Á.	2014	Hungary	Caucasian	234	225	12	25	141	23	10	164
Cascorbi, I.	2013	Germany	Caucasian	71	81	1	1	28	2	1	30
Magliulo, L.	2011	Italy	Caucasian	54	285	0	17	22	3	37	120
Frankfort, S.V.	2006	Netherlands	Caucasian	48	41	3	0	43	1	1	33

**Table 4:** Meta-analysis of the ABCB1 1236C > T-2677G > T/A-3435C > T haplotypes and AD risk.

Haplotype	OR	95% CI	P	I^2^
CTT	0.58	0.31–1.08	0.09	0
TTC	1.99	1.24–3.18	0.00	0
TTT	0.90	0.56–1.44	0.65	55.0
